# Accuracy and tracing time of cephalometric analyses on a tablet or desktop computer: A prospective study

**DOI:** 10.1186/s13005-024-00413-5

**Published:** 2024-02-12

**Authors:** Moritz Kanemeier, Claudius Middelberg, Thomas Stamm, Felix Albert, Ariane Hohoff, Jonas Q. Schmid

**Affiliations:** 1 Department of Orthodontics, University of Münster, Albert-Schweitzer-Campus 1, 48149 Münster, Germany; 2 Institute of Biostatistics and Clinical Research, University of Münster, Schmeddingstr. 56, 48149 Münster, Germany

**Keywords:** Lateral cephalogram, Cephalometry, Cephalometric analysis, Radiograph, iPad, Tablet computer, Orthodontic education

## Abstract

**Background:**

This prospective study aimed to evaluate the influence of the computer type (tablet or desktop) on accuracy and tracing time of cephalometric analyses.

**Methods:**

Dental students used a web-based application specifically developed for this purpose to perform cephalometric analyses on tablet and desktop computers. Landmark locations and timestamps were exported to measure the accuracy, successful detection rate and tracing time. Reference landmarks were established by six experienced orthodontists. Statistical analysis included reliability assessment, descriptive statistics, and linear mixed effect models.

**Results:**

Over a period of 8 semesters a total of 277 cephalometric analyses by 161 students were included. The interrater reliability of the orthodontists establishing the reference coordinates was excellent (ICC > 0.9). For the students, the mean landmark deviation was 2.05 mm and the successful detection rate for the clinically acceptable threshold of 2 mm suggested in the literature was 68.6%, with large variations among landmarks. No effect of the computer type on accuracy and tracing time of the cephalometric analyses could be found.

**Conclusion:**

The use of tablet computers for cephalometric analyses can be recommended.

## Background

The analyses of lateral cephalograms are a fundamental part of orthodontic diagnostics and treatment planning. They are used to determine the skeletal, dental and soft tissue relations, to evaluate treatment effects and to assess the vertebrae [1–7]. For this purpose, defined landmarks are placed on the radiographs. These can be anatomical, radiological or constructed points. Parameters such as angles, distances or ratios are measured between these landmarks and compared to standard values.

Standardized cephalometric radiographs were introduced into orthodontics by Broadbent and Hofrath in 1931 [8, 9]. The 22-item analysis used today in the Department of Orthodontics at the University of Münster is based on the analyses by Downs, Ricketts, Rakosi and Steiner [1, 2, 10, 11].

Originally, the analyses were performed manually by drawing the landmarks, angles, and distances on the analog lateral cephalogram by hand [12]. The greatest potential for error has always been in the localization of the landmarks [13]. As early as in the 1960s, computer-based systems were developed with the intention to enable faster and less error-prone cephalometric analyses. Landmark coordinates were initially transferred by hand using a drawing table [14] and later using digital reading systems [15], which were only slowly gaining acceptance due to their high cost [16]. Meanwhile, methods for digitizing radiographs were already developed [17], but until the 1990s these were qualitatively inferior to the use of digital readout systems [18]. Nowadays, direct digital x-ray technology eliminates the need for time-consuming and quality-reducing intermediate steps for viewing and tracing cephalograms on a computer. Furthermore, digital radiographs offer the option of changing the image in contrast, brightness and size, so that structures of different translucency can be viewed in detail. Another advantage of digital x-ray technology is the lower radiation dose for the patient [19].

For the diagnosis of digital radiographs a darkened room and a suitable viewing monitor are required. The use of tablet computers for radiographic analysis was already considered shortly after the introduction of the iPad (Apple, Cupertino, CA, USA) in 2010 [20]. Initial comparisons to conventional liquid-crystal displays (LCD) [21, 22] were promising, but the observer performance on iPads was found to be significantly lower than with calibrated monitors [23]. With the introduction of the high-resolution “Retina” display as part of the third-generation iPad in 2012, there was no longer a significant difference in comparison to calibrated viewing monitors [24] and the American Board of Radiology considered the iPad’s retina display adequate for examination in all specialties [25]. There was also no significant difference between tablet computers and viewing monitors in terms of reliability of landmark identification [26]. Finally, a 2015 systematic review found that the use of a tablet computer does not generally affect the interpretation of a radiograph [27].

In contrast to a PC with a viewing monitor, the use of a tablet computer allows for more flexible work. One can perform analyses directly in a darkened lecture hall, and even patient-side use is an option, since sterile packaging and disinfection of the device are possible [28, 29].

When using a tablet computer, inputs are made with the finger or a stylus directly on the touchscreen. The reproducibility of cephalometric analyses on tablet computers using a stylus and desktop computers using a mouse driven cursor has been studied previously, and no differences in measurements between the two modalities were found for any of the cephalometric parameters [30].

The aim of this study was to investigate the accuracy and tracing time of dental students when identifying landmarks on lateral cephalograms using a tablet or desktop computer. The null hypothesis was that the device used would have no effect on the accuracy or tracing time of landmark identification.

## Methods

This prospective study received approval from the Ethics Commission of the Medical Faculty of the University of Münster, Germany (2021-060-f-S). The study took place at the Department of Orthodontics at the University Hospital Münster, Germany.

### Software

A web-based application for performing cephalometric analyses of digital lateral cephalograms was developed.

The application was implemented with Typescript using the React frontend framework. Internationalisation for German and English was realised using the react-intl library to allow for future international use of the software. To import radiographs according to the Digital Imaging and Communications in Medicine (DICOM) standard, a lightweight parser was implemented.

**Fig. 1:**
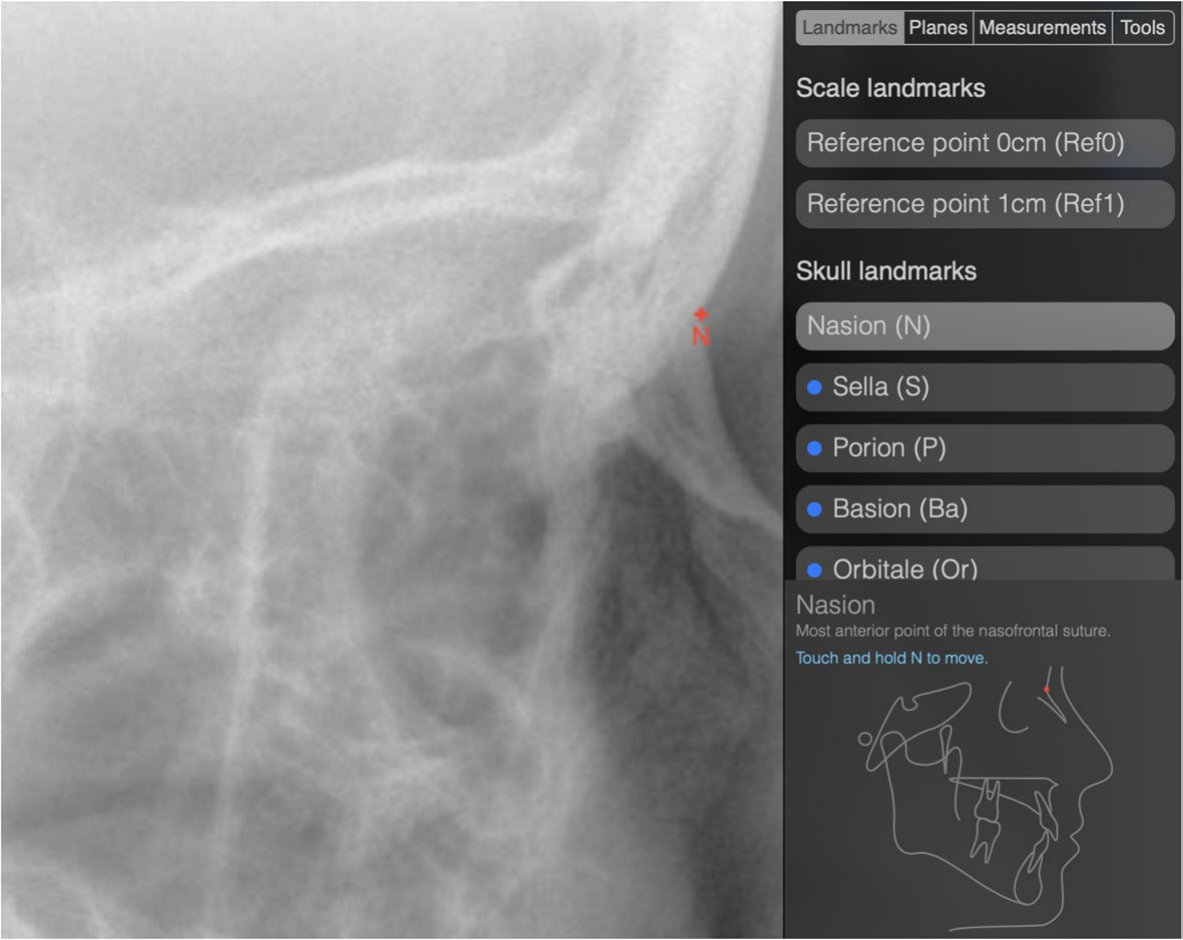
Placement of landmark Nasion in the web-based application. A small schematic drawing at the lower right edge shows the examiner the ideal position of the selected landmark and its definition

The application allows the brightness, contrast and magnification of the cephalogram to be freely adjusted. The sequence in which the landmarks are placed is suggested by a list representation, but is freely choosable. Placed landmarks can be corrected at any time. To assist the examiner, a small schematic drawing showing the ideal position of the selected landmark and its definition is provided (Fig. 1, Table 1).

**Table 1:** Definitions of the Landmarks used in the 22-item cephalometric analysis of the University of Münster as shown in the software

Landmark	Definition
S	Center of the bony sella turcica crypt.
N	Most anterior point of the nasofrontal suture.
P	Upper border of the external acoustic meatus.
Ba	Lowest point of the occipital bone at the anterior border of the foramen occipitale magnum in the median sagittal plane.
Or	Lowest point of the bony margin of the orbit.
Pt	Intersection of the lower boundary of the foramen rotundum with the posterior margin of the pterygopalatine fossa.
Spp	Most posterior point of the horizontal part of the palatine bone.
Spa	Anterior tip of the anterior nasal spine. Lowest and most anterior point of the piriform aperture.
A	Most dorsal point of the anterior curvature of the maxilla between the alveolar process and Spa.
Co	Highest point on the head of the condylar process.
DC	Midpoint of the condylar process on the [BaN] line.
R1	Lowest point of the curvature.
R2	Constructed parallel to FH.
R3	Lowest point.
R4	Constructed perpendicular to FH.
Xi	Center of the ramus of the mandible.
hT	Most caudal point of the body of the mandible.
Me	Caudal point of the outer contour of the symphysis.
Po	Most ventral point of the bony chin.
B	Lowest point of the outer contour between the mandible base and the alveolar process.
Pm	Inflection point between B and Po.
Gnk	Intersection of MP (hT-Me) and FP (N-Po).
UpIe	Most anterior point of the upper incisal edges.
UpIa	Apex of the most anterior upper central incisor.
LoIe	Most anterior point of the lower incisal edge.
LoIa	Apex of the most anterior lower central incisor.
1UpMdc	Distal contact point of the first upper molar.
1UpMma	Apex of the mesial root of the first upper molar.
Ap	Most anterior point of the nose.
Sn	Most posterior superior point of the nasolabial curvature.
UpL	Point at the junction of philtrum and upper lip.
LoL	Most anterior point of the lower lip.
Pom	Most ventral point of the chin contour.

The same web-based application was used on both the tablet and desktop computers. Therefore, a software-independent comparison of the cephalometric analysis performed with the two types of computers was possible.

To carry out the analyses, each student was provided with an iPad with Retina display (Apple, Cupertino, CA, USA) while the students used their own desktop computers.

### Data acquisition

Of all lateral cephalograms taken at the Department of Orthodontics in 2012-2017, 30 were randomly selected using a random number generator [31]. To obtain the radiographs, the heads of all patients were aligned with the sagittal plane perpendicular to the X-rays and the Frankfurt plane parallel to the floor. The teeth were in maximum intercuspation and the lips closed. After anonymisation of the cephalograms the following exclusion criteria were applied: unerupted or missing incisors, unerupted or missing first molars, malposition of the head in the cephalostat, osteosynthesis plates in situ or a missing scale. Selection was made without regard to gender, type of occlusion or skeletal pattern. After application of the exclusion criteria, 26 radiographs remained. From these, three were finally selected using the random number generator.

**Fig. 2:**
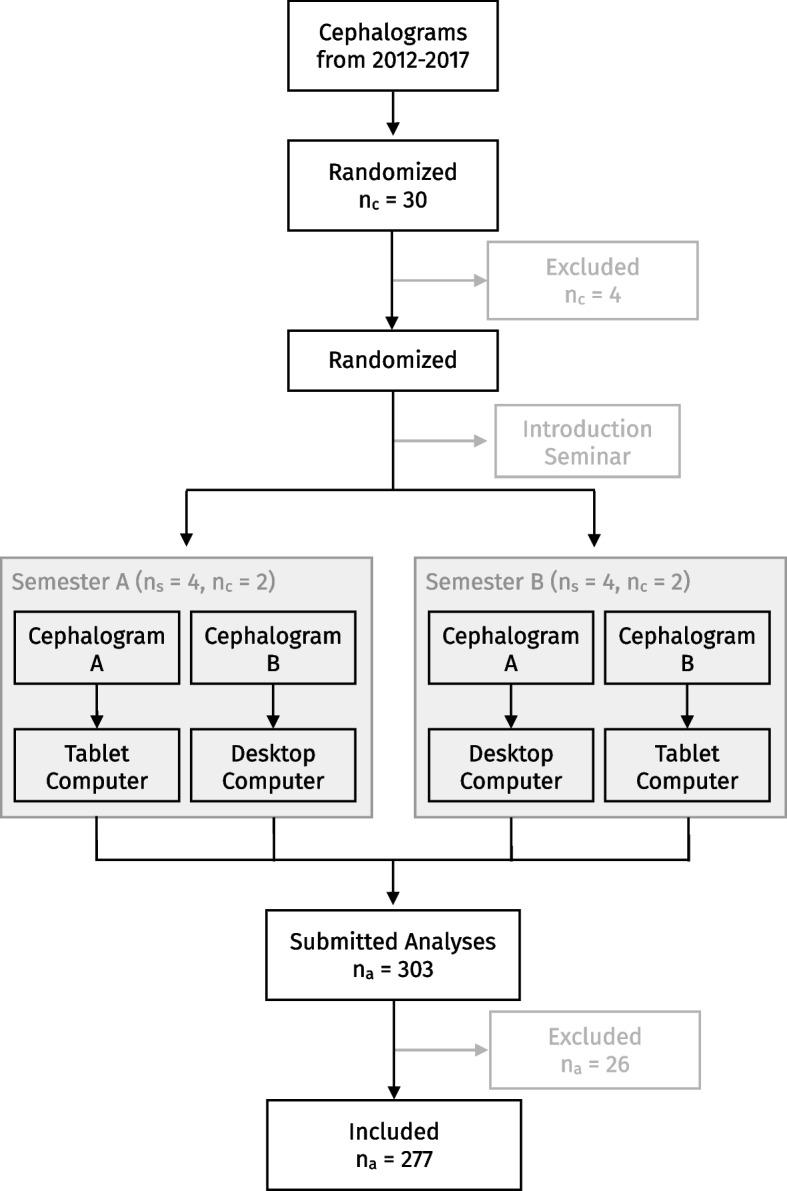
Selection and allocation of the cephalograms with number of cephalograms ($n_c$), semesters ($n_s$), and analyses ($n_a$)

One cephalogram was used to introduce the software to the students only. The other cephalograms (A, B) were analyzed by the students on the tablet and desktop computer accordingly. Two different cephalograms were used to avoid learning effects. The assignment of the cephalograms (A, B) to the computer type (tablet, desktop) was switched semester wise so that an influence of the cephalogram could be assessed separately from an influence of the device (Fig. 2).

**Fig. 3:**
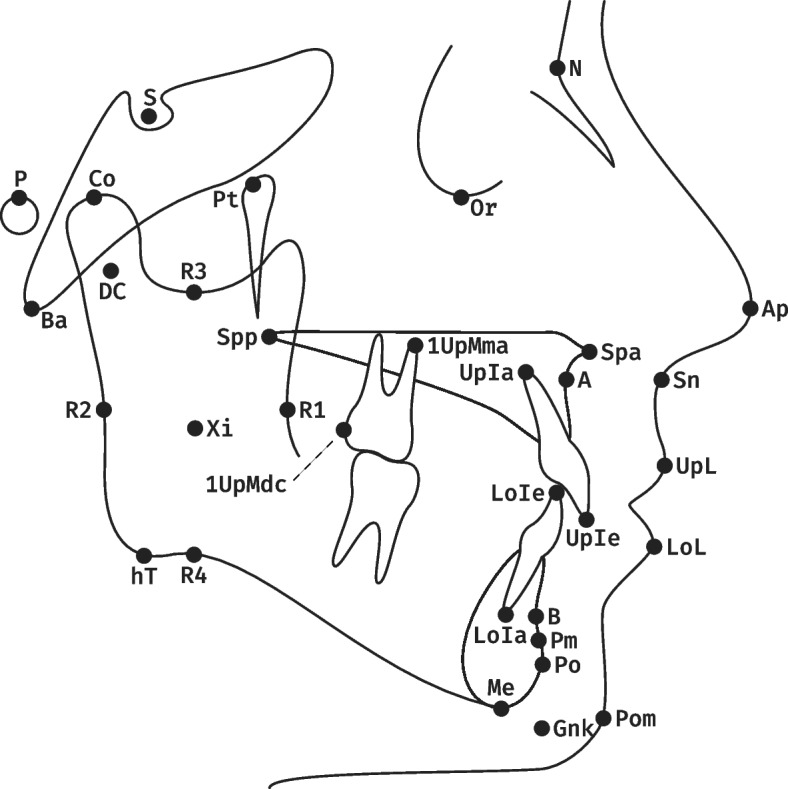
Landmarks used in the 22-item cephalometric analysis of the University of Münster: Nasion (N), Basion (Ba), Orbitale (Or), Porion (P), Pterygoid point (Pt), Sella (S), Anterior nasal spine (Spa), Posterior nasal spine (Spp), A point (A), Condylion (Co), Condylar midpoint (DC), Anterior border of the Ramus (R1), Posterior border of the Ramus (R2), Semilunar incisure (R3), Lower border of the Ramus (R4), Ramus midpoint (Xi), Horizontal tangent point (hT), Menton (Me), Pogonion (Po), B Point (B), Suprapogonion (Pm), Constructed gnathion (Gnk), Upper Incisor edge (UpIe), Upper Incisor apex (UpIa), Lower Incisor edge (LoIe), Lower Incisor apex (LoIa), First Upper Molar mesial apex (1UpMma), First Upper Molar distal contact (1UpMdc), Apex nasi (Ap), Subnasal (Sn), Upper Lip (UpL), Lower Lip (LoL), Pogonion molle (Pom). Figure adapted from [32]

Eligible participants were dental students of one orthodontic course that is part of the clinical curriculum in the seventh semester at the University of Münster. All students received the same education on cephalometric anaylsis. The course consisted of a lecture on the history, landmarks, planes and measurements of cephalometry combined with practical exercises on manual landmark positioning. The course lasts four hours and is divided into five sessions. This is followed by a 45-minute software demonstration session. The cephalograms used in this study were not used in the teaching or during the demonstration to avoid a learning effect.

Each student performed the 22-item cephalometric analysis of the University of Münster on the tablet computer (using a finger) and desktop computer (using a mouse) in no particular order. The students were instructed to perform the analysis without interruption and in a darkened room. The landmarks used for the 22-item analysis can be found in Fig. 3.

The landmark locations as well as timestamps for the first and last landmark placement were exported from the software in JSON (JavaScript Object Notation) format and submitted for evaluation. The JSON files were pseudonymised and processed using a Python script. The pseudonym was generated from the plain name and a salt (a random string) using the cryptographic one-way function SHA3-256 and subsequent sorting and ranking.

Exclusion criteria for the submitted cephalometric analyses were use of a cephalogram other than the ones provided, incorrect assignment of the cephalogram to the device type, missing landmarks, and duplicate submissions.

To establish a reference, six experienced orthodontists performed the analysis for each image on a desktop computer with the calibrated medical viewing monitor RadiForce RX220 (EIZO, Hakusan, Ishikawa, Japan) in a darkened room. Mean values for each landmark position were used as the reference ($x_{i_{ref}}$, $y_{i_{ref}}$).

Students’ accuracy was evaluated as the mean radial error (MRE) in mm (Eqs. 1 and 2), defined as the sum of all Euclidean distances ($d_i$) to the reference landmarks divided by the number of landmarks ($l=33$).

1 $$\begin{aligned} d_i = \sqrt{\left(x_{i_{stud}} - x_{i_{ref}}\right)^2 + \left(y_{i_{stud}} - y_{i_{ref}}\right)^2} \end{aligned}$$

2 $$\begin{aligned} MRE = \frac{\sum _{i=1}^{l}{d_i}}{l} \end{aligned}$$

Timestamps of the placement of the first and last landmark were recorded and the difference used as a measure of the students tracing time.

The resulting dataset contained the pseudonym of the student, the identifier of the image (A or B), the computer type used (tablet, desktop), the order identifier (0 if this is the students first analyses, 1 otherwise), the time required for identification of all landmarks in minutes and the students accuracy as defined above.

### Statistical analysis

The reliability of the established reference coordinates was assessed with an intraclass correlation coefficient using a two-way mixed effects model for the absolute agreement of multiple raters (ICC(A,k) according to McGraw and Wong [33]) using the irr package [34] for R [35]. The level of reliability was defined according to Koo and Li [36]: poor reliability $<0.5$, moderate reliability $<0.75$, good reliability $<0.9$, excellent reliability $>0.9$.

Descriptive statistics were performed for the students accuracy, tracing time and successful detection rate. A deviation of 2 mm was considered clinically acceptable [37, 38].

Linear mixed effect analysis was performed to test the influence of the device on accuracy and tracing time. Computer type (tablet or desktop), cephalogram (A or B), gender of the student, and order of analysis were considered as fixed effects. A random intercept for subjects was also included. The significance of each fixed effect was tested by a likelihood ratio test of a model with that effect against a null model. In a second step, models with an increasing number of these significant effects were tested against the previous models. Finally, a model with all effects that showed a significant improvement was selected. The linear mixed effects analyses were executed using the lme4 package [39] for R [35].

## Results

The study was conducted from 2018 to 2022 over a period of 8 semesters. During this period 303 analyses were submitted. Of these 26 analyses had to be excluded due to the exclusion criteria: 16 contained the wrong cephalogram, 5 had a screenshot of the provided cephalogram, 3 had missing landmarks, and 2 were invalid json files, ultimately resulting in 277 submissions with a total of 9141 landmarks being included in the study. The resulting study group consisted of 161 (108 female, 53 male) students.

The interrater reliability of the six orthodontists that established the reference coordinates (Table 2) was excellent ($ICC~>~0.9$).

**Table 2:** Reference coordinates for the landmarks as established by six orthodontists with the corresponding interrater reliabilities

	Image A			Image B		
Landmark	Coordinate (x, y)		ICC	Coordinate (x, y)		ICC
S	588.88	152.56	1	360.58	217.19	1
N	934.58	126.09	1	824.02	179.64	1
P	468.58	220.84	1	197.60	313.30	0.99
Ba	453.87	309.68	1	192.49	473.38	1
Or	853.62	256.17	1	703.35	375.07	1
Pt	666.47	223.62	1	463.15	328.32	1
Spp	685.18	339.47	1	485.33	494.78	0.99
Spa	925.97	379.03	1	844.84	522.46	1
A	908.00	389.21	1	796.58	550.87	1
Co	526.21	247.91	1	271.20	342.25	0.99
DC	537.95	277.44	1	280.59	432.39	1
R1	681.03	385.09	1	467.46	568.39	0.99
R2	542.76	372.39	1	260.77	542.93	1
R3	602.15	311.73	1	374.88	479.39	0.99
R4	585.33	494.85	1	350.11	715.36	0.98
Xi	609.52	404.73	1	361.74	598.12	0.98
hT	599.22	502.05	1	344.90	769.04	1
Me	828.56	609.04	1	698.74	868.92	1
Po	860.81	580.67	1	736.28	830.39	1
B	864.70	527.11	1	727.95	756.28	0.99
Pm	862.51	556.61	1	734.57	796.98	1
Gnk	854.28	621.05	1	729.89	877.71	1
UpIe	914.55	480.74	1	825.77	683.21	1
UpIa	883.65	376.10	1	756.35	550.53	1
LoIe	895.17	452.82	1	771.70	638.02	1
LoIa	834.90	527.52	1	682.84	760.65	1
1UpMdc	738.83	413.65	1	542.13	600.51	1
1UpMma	799.05	353.53	1	635.35	515.25	1
Ap	1058.36	337.83	1	993.68	469.87	1
Sn	993.00	396.88	1	909.98	550.54	1
UpL	979.41	460.86	1	913.15	636.74	1
LoL	956.21	497.29	1	870.12	715.84	1
Pom	927.67	596.93	1	792.92	847.70	1

### Accuracy of students’ landmark identification

**Fig. 4:**
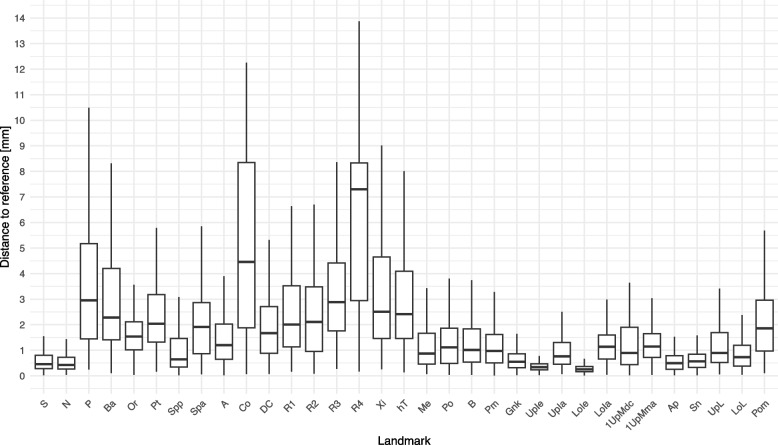
Deviation of the students’ landmarks to the reference in mm

**Fig. 5:**
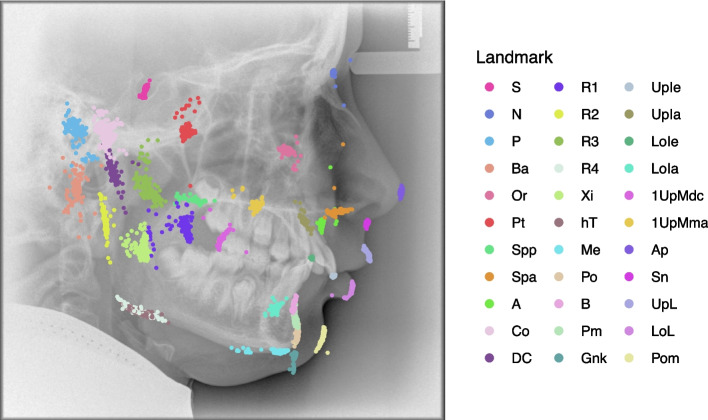
Positioning of the landmarks by the students on image B

The mean landmark deviation of the students was 2.05 mm (SD = 2.63). The landmarks LoIe, UpIe, Ap, Sn, S and N were identified with the smallest deviation. The largest deviation was found for the landmark R4, Co, R3, P, Ba and R1. The deviations for all landmarks are listed in Table 3 and visualised in Fig. 4. The landmarks as placed by the students are shown in Fig. 5.

**Table 3:** Accuracy of students’ landmark identification evaluated as the mean radial error and the successful detection rate below different thresholds

	Mean Radial Error (mm)			Successful Detection Rate (%)			
Landmark	M	SD	95% CI	1 mm	2 mm	4 mm	8 mm
S	0.79	1.07	[0.67, 0.92]	81.9	90.3	99.6	99.6
N	0.81	1.24	[0.66, 0.96]	80.9	92.4	96.8	99.6
P	3.77	3.04	[3.41, 4.13]	15.9	31.4	65.0	89.5
Ba	3.76	3.69	[3.32, 4.20]	14.8	42.6	70.0	85.2
Or	1.77	1.29	[1.62, 1.92]	24.5	71.1	94.2	99.6
Pt	3.18	3.42	[2.78, 3.59]	13.4	47.3	78.3	90.6
Spp	1.39	3.41	[0.99, 1.80]	64.6	83.0	92.8	99.6
Spa	2.58	3.80	[2.13, 3.02]	26.7	53.4	85.9	96.0
A	1.58	1.63	[1.38, 1.77]	40.4	74.4	94.6	99.6
Co	4.92	3.26	[4.54, 5.31]	11.2	27.4	45.8	72.9
DC	2.53	2.81	[2.20, 2.87]	28.5	58.8	84.1	93.1
R1	3.50	3.96	[3.03, 3.97]	18.4	48.0	76.9	87.4
R2	2.76	2.48	[2.46, 3.05]	26.7	45.8	80.5	94.6
R3	3.96	3.41	[3.56, 4.36]	6.5	30.7	69.0	88.8
R4	5.86	3.30	[5.47, 6.25]	10.1	18.4	36.1	65.0
Xi	3.19	2.16	[2.94, 3.45]	13.4	38.3	63.5	98.2
hT	2.89	1.92	[2.66, 3.12]	17.0	39.0	74.0	97.5
Me	1.78	2.81	[1.45, 2.11]	56.3	78.7	90.6	96.0
Po	1.29	0.96	[1.17, 1.40]	46.2	80.9	98.2	100.0
B	1.36	1.17	[1.22, 1.50]	49.5	77.6	96.8	99.6
Pm	1.19	0.98	[1.07, 1.30]	50.9	84.1	99.3	100.0
Gnk	0.81	0.96	[0.69, 0.92]	79.8	93.9	97.1	100.0
UpIe	0.37	0.19	[0.35, 0.39]	99.6	100.0	100.0	100.0
UpIa	1.11	1.08	[0.98, 1.24]	61.7	88.1	97.5	99.6
LoIe	0.28	0.16	[0.26, 0.30]	99.6	100.0	100.0	100.0
LoIa	1.30	0.98	[1.19, 1.42]	43.0	84.5	97.5	100.0
1UpMdc	1.69	2.52	[1.39, 1.99]	53.4	77.3	91.3	97.5
1UpMma	1.60	2.18	[1.34, 1.86]	40.4	84.8	96.0	96.8
Ap	0.61	0.47	[0.55, 0.66]	83.0	98.9	100.0	100.0
Sn	0.67	0.58	[0.60, 0.74]	83.8	97.5	99.3	100.0
UpL	1.31	1.15	[1.17, 1.44]	54.2	78.7	96.8	100.0
LoL	0.94	0.86	[0.84, 1.05]	67.1	92.8	99.3	100.0
Pom	2.05	1.37	[1.88, 2.21]	27.8	54.5	91.3	99.6

The likelihood ratio tests showed a significant effect of the image (${\chi }^2(1)~=~19.10$, $p~<~.001$) and students’ gender (${\chi }^2(1)~=~5.54$, $p~=~0.02$) on the accuracy. The type of computer (${\chi }^2(1)~=~0.98$, $p~=~0.32$) and the order in which the analyses were conducted (${\chi }^2(1)~=~0.11$, $p~=~0.75$) had no significant effect. There was no significant interaction between image and gender (${\chi }^2(1)~=~0.08$, $p~=~0.78$).

The resulting model suggested that image B was more difficult to analyse than image A, with an estimated effect of 0.21 mm, and that male students performed better than female students regardless of the image, with an estimated effect of 0.24 mm. The estimates and confidence intervals of the effects are shown in Table 4.

**Table 4:** Linear mixed effect model for accuracy (deviation in mm) and tracing time (in minutes per analysis)

Response	Predictor	Estimate	95% CI	p
Accuracy	Intercept	2.04	[ 1.92, 2.16 ]	$p~<~.001$
	Image (B)	0.21	[ 0.12, 0.29 ]	$p~<~.001$
	Gender (male)	-0.24	[ -0.43, -0.05 ]	$p~=~0.02$
Time	Intercept	23.79	[ 20.40, 27.17 ]	$p~<~.001$
	Order (2nd)	-11.72	[ -16.68, -6.76 ]	$p~<~.001$

#### Successful detection rate

The successful detection rate (SDR) for the clinically acceptable threshold of 2 mm was 68.6% over all landmarks. The SDR for 2 mm was greater than 90% for 8 landmarks and less than 35% for 4 landmarks. The SDRs for all landmarks and different thresholds are listed in Table 3.

### Tracing time

The median tracing time for the students was 11.80 minutes per analysis (IQR 7.70–20.49), while for the orthodontists it was 5.15 minutes (IQR 4.27–7.24).

Regarding students’ tracing time the likelihood ratio tests showed a significant effect of the order in which the analyses were conducted (${\chi }^2(1)~=~19.55$, $p~<~.001$). The image (${\chi }^2(1)~=~0.08$, $p~=~0.77$), type of computer (${\chi }^2(1)~=~1.53$, $p~=~0.22$) and gender (${\chi }^2(1)~=~0.03$, $p~=~0.86$) had no significant effect.

The resulting model suggests that performing the analysis for the second time is faster with an estimated effect of 11.72 minutes. The estimates and confidence intervals of the effect are shown in Table 4.

### Cephalometric measurements

Cephalometric measurements were calculated using both the reference landmarks and the landmarks placed by the students. Significant differences were only found for four of the 22 measurements (facial depth, mandibular plane, relative mandibulary length and relative maxillary length) as shown in Table 5.

**Table 5:** Cephalometric measurements calculated from the reference landmarks and those placed by the students. Descriptive statistics with mean (M) and standard deviation (SD) as well as the results of t tests (assuming heterogeneous variances)

	Reference		Students		
Measurement	M	SD	M	SD	p
Facial axis (^∘^)	94.41	1.63	94.32	3.75	0.90
Facial depth (^∘^)	86.03	0.41	85.08	2.87	<0.00
SNB (^∘^)	75.74	0.53	75.58	1.45	0.54
Mandibular plane (^∘^)	19.76	0.63	21.02	3.12	<0.00
Inner gonion angle (^∘^)	150.38	4.14	151.32	5.98	0.61
Rel. mand. length (mm)	96.31	1.24	100.63	3.86	<0.00
Maxillary position (^∘^)	63.33	1.37	62.81	3.10	0.43
SNA (^∘^)	79.84	1.24	79.96	2.55	0.84
Palatal plane (^∘^)	-4.09	0.67	-3.35	3.40	0.08
Rel. max. length (mm)	83.09	1.54	86.56	3.16	<0.00
Lower facial height (^∘^)	35.60	1.06	36.16	2.00	0.27
Convexity of point A (mm)	3.25	0.90	3.64	1.66	0.35
Rel. max./mand. length	1.17	0.05	1.17	0.05	0.96
Lower incisor position (mm)	0.55	0.61	0.46	1.17	0.76
Lower incisor inclination (^∘^)	25.00	1.43	24.41	2.99	0.39
Upper incisor position (mm)	5.76	0.56	5.44	0.97	0.24
Upper incisor inclination (^∘^)	30.30	0.68	30.85	2.58	0.15
Inter incisor angle (^∘^)	124.70	1.16	124.74	2.63	0.94
Vertical molar distance (mm)	-0.94	1.30	-0.66	2.18	0.64
Sagittal molar distance (mm)	18.26	3.17	19.16	5.13	0.53
Lower lip to E-line (mm)	-3.96	0.35	-3.92	0.42	0.84
Upper lip drape (^∘^)	83.25	0.87	82.68	3.50	0.24

## Discussion

The present study focuses on the development and evaluation of a web-based application for performing cephalometric analyses of digital lateral cephalograms. The study results showed no influence of the type of computer (i.e. tablet or desktop) on the students’ accuracy or speed when performing the analysis.

Previous studies on app-based versus manual tracing showed no clinically relevant differences in tracing accuracy [40–42]. Recent studies comparing desktop computers to smartphones found comparable results on tracing accuracy [43, 44], but inconsistent results on tracing time [44, 45]. For tablet computers with pen-input, two studies found no significant difference from desktop-computer-based analyses [30, 46] and one study found that the mobile apps were inferior [47]. To our knowledge, there have been no studies comparing computers with touch-input (i.e. smartphone or tablet) with desktop computers, using the same application on both devices.

Most studies comparing the accuracy of tracing methods [30, 41–45, 47] used the cephalometric measurements as a measure of tracing accuracy, while one study from 2015 [46] as well as more recent studies covering neural network based approaches used the landmark location.

The advantage of using landmark locations is that they are easier to compare across studies, as the number of different - non-comparable - measurements that can be made with the same set of landmarks is naturally greater. In addition, angular measurements in cephalometry mask placement errors that occur when the landmark is misplaced along the arms of the measured angle.

The landmarks identified with the smallest deviation (LoIe, UpIe, Ap, Sn, S and N) are consistent with previous studies on the reliability of cephalometric landmarks [18, 48–57]. Regarding large deviations, the results are also in agreement with previous studies stating that the identification of landmarks in the petrous temporal region (i.e. Ba, Co and P) is difficult due to superimpositions and that the error is generally larger for landmarks along gradually curved surfaces (i.e. R1, R3 and R4) due to elliptical error distribution [53].

The results of the mixed linear effect model showed that image B was slightly more difficult to analyze, with an increase in mean deviation of 0.2 mm. This could be explained by more structures being superimposed in image B. It was also found that the gender of the students had a significant influence, with male students being more accurate by 0.2 mm.

In the study population, the gender distribution was unbalanced with 108 female and 53 male students. This imbalance is related to the higher prevalence of female students in dental education. In recent decades, the proportion of female students in dentistry has increased, which can be attributed to a higher application rate with comparable admission rates between the genders [58]. Considering the unequal gender distribution and the small effect size found, the gender-specific difference in accuracy should be interpreted with caution.

Regarding the tracing time, the results showed that the students perform the second analysis faster than the first one with a mean decrease of 11.72 minutes, indicating a learning effect. The fact that the students needed a median of 12 minutes for a cephalometric analysis, while the orthodontists were significantly faster with a median of 5 minutes, shows that the time needed decreases with increasing experience. The other effects considered (i.e. device and gender) had no significant influence on the tracing time.

To assess the clinical performance of the students, cephalometric measurements were calculated for both the reference and student landmarks (Table 5). The variability of the student measurements were comparable to that reported in previous studies ([41–45, 47]). A significant difference to the reference was only found for four of the 22 measurements (facial depth, mandibular plane, relative mandibular length and relative maxillary length).

In view of the progress made in the field of automated cephalometry, the question arises as to whether manual landmark positioning is still relevant. Although there has been great progress in the field of automated evaluation of cephalometric analyses in recent years with the availability of open annotated datasets [59] and the continuous development of various neural network architectures [60], recent studies that have evaluated cephalometric analyses by such AI-based systems and those performed by experienced orthodontists could only recommend the use of these systems under supervision [61]. On the other hand, the idea of collaboration between AI-based systems and students seems promising [62] and should be evaluated as an approach to support the teaching of cephalometry.

According to our results, using tablets for cephalometric analyses in orthodontic education must be considered an appropriate approach and can be recommended. Considering that teaching cephalometric landmark identification with a smartphone-based application has been shown to be at least equivalent to lecture-based instruction [63], a fully digital workflow seems feasible.

### Strengths and limitations

The prospective nature and the large number of submitted cephalometric analyses can be seen as a strength of the present study. It provides valuable data on what can be expected from beginners in orthodontics in terms of accuracy and tracing time. However, this study has some limitations that need to be considered when interpreting the results.

The cephalograms chosen seemingly had different degrees of difficulty. The analysis of multiple cephalograms was conducted to minimise bias in the results with respect to landmarks that are particularly difficult to locate in the image. Due to the voluntary nature of the participation, the analysis of only two cephalograms per student was possible, resulting in a limited sample size. A larger sample size would increase the generalisability of the findings and provide more statistical power.

The study was conducted at the Department of Orthodontics at the University Hospital Münster, Germany. The findings may not be applicable to other universities with different curricula, as there may be variations in the expertise and techniques employed at different institutions.

The students who performed the cephalometric analyses were aware of the computer type (tablet or desktop) they were using. This lack of blinding could introduce bias and influence their performance.

The students were instructed to conduct the analysis without interruption and in a darkened room, but this could not be controlled and should be taken into account when interpreting the results. In addition, the timestamps were only registered for the entire session and not for individual landmarks, since the order of landmark placement and later corrections of their position could not be tracked.

Each student was provided with an iPad (Apple, Cupertino, CA, USA) while the students used their own desktop computers, which must be considered as another limitation of the present study, as it contributes to the heterogeneity of the desktop computer based analyses, also because it could not be guaranteed that the respective screens were suitable for x-ray diagnosis.

The overall accuracy of the students was low and the tracing time was high, which was to be expected as the students were taught cephalometry in the semester in which the study was conducted.

The study focused on the students’ accuracy and tracing time as outcome measures. While these measures provide insights into the performance of the web-based application, its clinical validity was not evaluated. Further research is required to identify and address any potential limitations introduced by the software itself and to assess its clinical validity.

## Conclusions

No significant influence of the device used to perform a cephalometric analysis was found with regards to accuracy and speed. The use of tablet computers for cephalometric analyses in orthodontic education can be recommended.

## Data Availability

The data presented in this study are available on reasonable request from the corresponding author.
